# Optimization of Impervious Surface Space Layout for Prevention of Urban Rainstorm Waterlogging: A Case Study of Guangzhou, China

**DOI:** 10.3390/ijerph16193613

**Published:** 2019-09-26

**Authors:** Huafei Yu, Yaolong Zhao, Yingchun Fu

**Affiliations:** 1 School of Geography, South China Normal University, Guangzhou 510631, Chinafuyc@m.scnu.edu.cn (Y.F.); 2 School of Resource and Environmental Sciences, Wuhan University, Wuhan 430079, China

**Keywords:** urban rainstorm waterlogging, impervious surfaces, optimization of spatial layout, ant colony optimization, Soil Conservation Service curve number model, Guangzhou

## Abstract

With the rapid expansion of impervious surfaces, urban waterlogging has become a typical “urban disease” in China, seriously hindering the sustainable development of cities. Therefore, reducing the impact of impervious surfaces on surface runoff is an effective approach to alleviate urban waterlogging. Presently, the development mode of many cities in China has shifted from an increase in urban scale to the improvement of urban quality through urban renewal, which is the current and future development path for most cities. Optimizing the design of impervious surfaces in urban renewal planning to reduce its impact on surface runoff is an important way to prevent and control urban waterlogging. The aim of this research is to construct an optimization model of impervious surface space layout under the framework of a geographic simulation technology-integrated ant colony optimization (ACO) and Soil Conservation Service curve number (SCS-CN) model (ACO-SCS) in a case study of Guangzhou in China. Urban runoff plots in the study area are divided according to the area of the urban planning unit. With the goal of minimizing the runoff coefficient, the optimal space layout of the impervious surfaces is obtained, which provides a technical method and reference for urban waterlogging prevention and control through urban renewal planning. The results reveal that the optimization of impervious surface space layout through ACO-SCS achieves a satisfactory effect with an average optimization rate of 9.52%, and a maximum optimization rate of 33.16%. The research also shows that the initial impervious surface layout is the key influencing factor in ACO-SCS. In the urban renewal planning stage, the space layout of the impervious surfaces with a high–low–high density discontinuous connection can be constructed by transforming medium-density impervious surfaces into low-density impervious surfaces to achieve the flat and long-type agglomeration of the low-density and high-density impervious surfaces, which can effectively reduce the influence of urban development on surface runoff. There is spatial heterogeneity of the optimal results in different urban runoff plots. Therefore, the policy of urban renewal planning for urban waterlogging prevention and control should be different. The optimized results of impervious surface space layout provide useful reference information for urban renewal planning.

## 1. Introduction

With the rapid development of urbanization, urban rainstorm waterlogging has become a typical “urban disease” in both developed and developing countries [1,2,3,4,5], and China serves as a prime example. According to the statistical results for 350 cities in China, 62% of these cities have suffered from urban rainstorm waterlogging especially in major cities such as Beijing, Shanghai, Nanjing, Tianjin, and Guangzhou [6,7,8,9,10,11]. Urban rainstorm waterlogging results in large losses to the social economy and to residential property, accordingly hindering the sustainable development of a city [12]. Eliminating urban rainstorm waterlogging risk has become an urgent mission for local governments, rainwater management agencies, and urban planning initiatives in such cities [13,14,15].

Many studies have indicated that waterlogging has a higher flood peak and shorter lag time in urban areas than in rural areas [16,17,18], which mainly resulted from the expansion of urban impervious surfaces [15,19,20,21] because rainwater infiltration on impervious surfaces is much lower than on pervious surfaces [22,23]. Drake et al. found that impervious pavement increases surface runoff by 43% [24]. When impervious surfaces account for more than 10%, the frequency of urban waterlogging will be greatly increased [25]. The increase in impervious surfaces will reduce the ability of rainwater infiltration [26], thereby increasing the surface runoff and peak flow, and eventually accelerating the occurrence of urban rainstorm waterlogging. However, impervious surfaces not only affect urban rainstorm waterlogging through the area, but also influence surface runoff through their spatial distribution [27,28,29]. Poff et al. found that different urban land use distributions have important effects on surface runoff [30]. Zheng et al. established four different urban spatial layouts in Ancaster, Ontario, Canada, and found that the influence of different urban spatial layouts on surface runoff was significantly different [31]. Mejia et al. used the annealing algorithm to simulate the expansion mode of impervious surfaces in different cities. The results show that the spatial layout of uniform expansion of low-density impervious surfaces has the least impact on urban rainstorm waterlogging [32]. Therefore, it was proven that impervious surfaces with the same areas make a great difference on surface runoff due to the variance in their spatial layout.

In summary, impervious surfaces through area and spatial layout simultaneously hinder the infiltration of rainwater, thus aggravating urban rainstorm waterlogging. Therefore, reducing the impact of impervious surfaces on surface runoff has become an important way to alleviate urban waterlogging. Currently, the mainstream practices in developed countries mainly include low-impact development (LID), best management practices, and green infrastructure [25]. These methods aim to reduce the impact of impervious surfaces on surface runoff by building permeable parking lots, semi-pervious roads, and highly pervious surfaces. Chinese scholars put forward the concept of a “sponge city”, aiming to reasonably construct a more pervious surface by means of ecological landscape science [33]. This shows that, at present, reducing the area of impervious surfaces is the main way to reduce its aggravated effect on waterlogging in various countries. However, due to the current development of cities, the large-scale reduction in urban impervious surfaces will seriously undermine the current urban structure [34,35,36,37]. Therefore, under the premise of protecting urban structures, reducing the influence of impervious surfaces on runoff from the perspective of spatial layout optimization is the key to the prevention and control of rainstorm waterlogging.

From Reform and Opening in 1978, China has experienced a rapid urbanization process [38]. At present, urban development has shifted from a simple scale increase to the improvement of stock quality, that is, the stage of urban renewal [39,40,41,42]. How to solve “urban diseases” such as rainstorm waterlogging by means of renewal planning is a new challenge in the field of urban renewal planning. Urban renewal is a planned reconstruction activity in the new development stage of the world’s major cities [43]. Therefore, under the premise of protecting the urban structure with the help of the urban renewal “embroidery”, how to reduce the impact of impervious surfaces on surface runoff from the perspective of spatial layout optimization, aiming to provide decision-making references for urban renewal planning for urban waterlogging prevention, will be the key breakthrough direction of this research.

Due to the successful application of urban land use optimization in economic geography, ecological geography, and social geography [44,45,46,47,48,49], reducing the negative impact of impervious surfaces on urban surface runoff from the perspective of spatial optimization is of great significance. The intelligent optimization algorithm realizes the explicit expression of spatial layout optimization of land use by virtue of the system dynamics mechanism based on a “bottom-up” perspective, from the micro mechanism to the macro expression. Relying on its fast and efficient spatial search ability, it has been applied in the research of spatial layout optimization of urban land use. Safarzadeh and Koosha solved the problem of multirow facility layout using a genetic algorithm [50]. Xu et al. used the Conversion of Land Use and its Effects at Small region extent to predict the land use layout of Guangzhou, China, for ecological service functions [51]. Jabir et al. used a heuristic ant colony algorithm to solve the multivehicle path scheduling for economy and emissions cost [52]. Ant colony optimization (ACO), as a typical representative of intelligent optimization algorithms, was first applied by Colorni et al. to solve the traveling salesman problem [53]. ACO has excellent search ability and efficient convergence ability, as well as efficient parallel computing ability and flexible scalability. In recent years, ACO has been widely used in the study of spatial layout optimization of urban land use [54,55,56]. This method avoids the complexity of rule design in the traditional behavior modeling process and helps to improve the efficiency of model construction and the accuracy of model simulation. Therefore, ACO was selected in this study for the optimization of impervious surface space layout.

Since surface runoff is the most intuitive expression of its influence on urban rainstorm waterlogging [8,57,58], the surface runoff coefficient is selected as the evaluation target of the optimization effect. Currently, surface runoff models are mainly calculated by the Streamflow Synthesis and Reservoir Regulation model, the Stanford model, the Tank model, the Sacramento model, the Storm Water Management Model, and the Soil and Water Assessment Tool [59,60,61,62,63,64]. However, due to the highly complex runoff generation process, which is dynamic and nonlinear and affected by many factors, these models require more input parameters and the modeling process is relatively complex. Soil Conservation Service curve number (SCS-CN) is a relatively mature model of surface runoff proposed by the US Natural Resources Conservation Service [65]. Characterized by simplicity, predictability, and stability due to its simplification of environmental factors affecting runoff generation, it is an effective tool for evaluating runoff in metropolitan areas without observed data and has been applied to different targets [66,67]. Yao et al. used SCS-CN to explore rainfall-runoff risk variance in different functional areas of Beijing, China [57]. Ansari et al. studied the spatiotemporal impact of urbanization on surface runoff by means of SCS-CN [68]. Kayet et al. coupled the Revised Universal Soil Loss Equation and SCS-CN to explore the relationship between rainfall and soil loss [69]. Considering the stability application of SCS-CN in various fields and its effective simulation of surface runoff in metropolitan areas, this model is selected as the surface runoff model for research.

In summary, the objective of this study is to construct an optimization model of impervious surface space layout integrated ACO and SCS-CN (ACO-SCS) to simulate the optimal space layout with the target of minimum surface runoff in a case study of Guangzhou. It will provide a reference to urban renewal planning agencies for urban waterlogging prevention and control.

The questions the study intends to answer are as follows:

(1) Can the optimization of impervious surface space layout reduce surface runoff? How can it be achieved?
(2) Does ACO-SCS based on geographic simulation technology realize the optimization of impervious surface space layout? What is the effect of optimization?
(3) How does the optimization result serve urban renewal planning for urban rainstorm waterlogging prevention?

## 2. Materials and Methods

**Figure 1:**
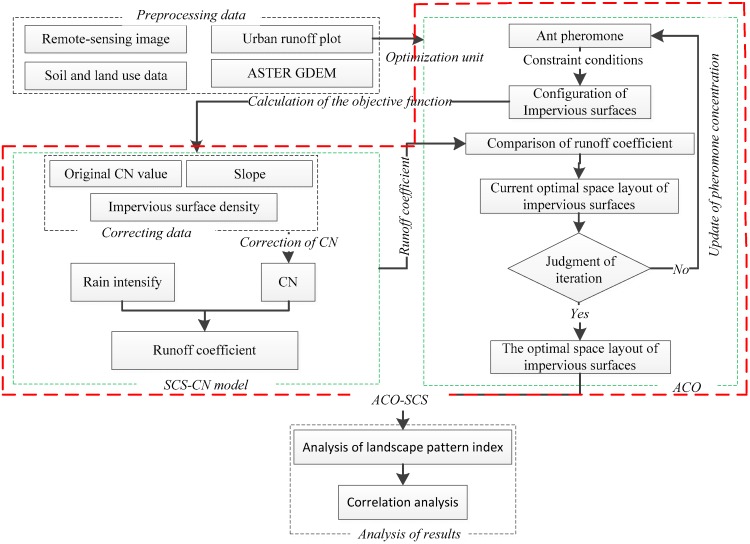
Integrated framework. Abbreviations: SCS-CN, Soil Conservation Service curve number; ACO, ant colony optimization; ASTER GDEM, Advanced Spaceborne Thermal Emission and Reflection Radiometer Global Digital Elevation Model.

The integrated framework of this research is shown in Figure 1. Firstly, data preprocessing was carried out to obtain impervious surfaces, slope, urban runoff plot, and initial CN value. Secondly, the ACO-SCS coupling model is built to design the probability function, heuristic function, adaptive function and pheromone concentration update function in the ACO. Then, the optimal combination of parameters is constructed, and the optimal impervious surface is obtained by running the model. Finally, the landscape pattern index analysis and correlation analysis are carried out according to the optimization results.

### 2.1. Study Area

**Figure 2:**
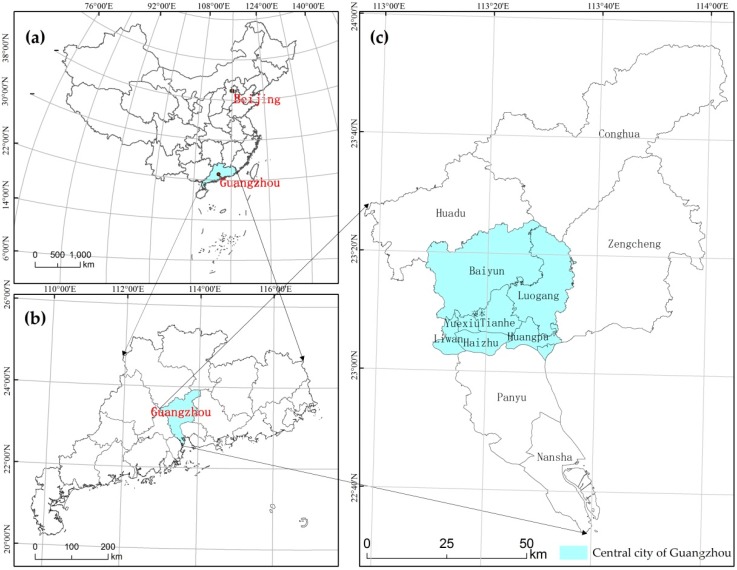
Study area: (**a**) location of Guangdong province in China; (**b**) Guangzhou’s location in Guangdong province; (**c**) central city of Guangzhou.

This research takes the central city of Guangzhou, China, as the study area. Guangzhou is the political, economic, scientific, educational, and cultural center of Guangdong province and an international business center (Figure 2). According to statistics, the gross domestic product (GDP) of Guangzhou reached 2.3 trillion yuan in 2018, fourth only to Shanghai, Beijing, and Shenzhen. Guangzhou is located between longitude 112°57′ and 114°3′ E and latitude 22°6′ and 23°56′ N. It is at the junction of the Xijiang, Beijiang, and Dongjiang Rivers, near the South China Sea. It has a maritime subtropical monsoon climate with high annual precipitation and heavy rain. Since the Reform and Opening more than 30 years ago, with the continuous expansion of the urban area of Guangzhou, urban rainstorm waterlogging disasters have occurred multiple times, causing large losses. According to statistics, from the 1980s to approximately 2010, the number of waterlogging events increased from 7 to 113 in 30 years, increasing nearly 16 times [70]. The affected area spread from the Yuexiu District in the central area to the rural-urban continuum, such as Tianhe, Haizhu, and Baiyun. Just in 2010, there were four serious urban rainstorm waterlogging events. In addition to climatic factors, important causes include the imperfect design of the urban pipe network and the unreasonable transformation of the terrain by urbanization [8,71,72]. Therefore, the frequent occurrence of rainstorm waterlogging in Guangzhou has a close relationship with urbanization [15,29].

### 2.2. Data and Preprocessing

Research shows that impervious surface expansion in Guangzhou slowed down in 2010 [73]. From 2000 to 2012, Guangzhou suffered the most severe urban rainstorm waterlogging [29]. It can be considered that 2010 is the key node for the impact of impervious surface expansion on urban rainstorm waterlogging. Therefore, this study collected remote sensing image data of Landsat7 Enhanced Thematic Mapper Plus with 30 m resolution in Guangzhou in October 2010. After radiometric calibration and atmospheric correction in the Environment for Visualizing Images) 5.0, the linear spectral mixture analysis was used to extract the impervious surfaces. The high-resolution image in 2010 was used to achieve sampling and classification verification. The final accuracy evaluation result of impervious surface extraction, with standard deviation 3.3%, mean absolute rrror 0.20, and root-mean-square rrror 0.26, meets the research requirements.

Advanced Spaceborne Thermal Emission and Reflection Radiometer Global Digital Elevation Model (ASTER GDEM) terrain data with 30 m resolution were used to extract the slope used to correct the CN value. In this study, soil data and land use data were used to extract the CN values in the SCS-CN. Soil data are global soil classification standard data with 1 km resolution based on the World Geodetic System-1984 (WGS84) and it is freely available for download from the China WestDC. Land use data are compiled from the second national land survey in Guangzhou with 1:2000 scale. This study used the urban catchment area divided by Li et al. as the optimization unit [74]. However, there were some topology errors in the initial urban catchment area, including long edge, gap, noncoincidence of boundary, and too small of a catchment area. After the above topology errors were corrected, the study area was divided into 154 urban runoff plots with the area ranging from 2.1 km^2^ to 101.5 km^2^.

Universal Transverse Mercator projection and WGS84 geodetic coordinates system were used in the study. All data details are shown in Table 1.

### 2.3. Optimization Model of Impervious Surface Space Layout

#### 2.3.1. SCS-CN

The hydrological model is a highly abstract simplification of the hydrological system [65,75]. SCS-CN has a low demand for parameters and can be applied to basins with limited data for accurately calculating the actual runoff of basins with different data. In view of the above advantages, SCS-CN is adopted as the objective function of the optimization model for calculating the surface runoff in this study. Simultaneously, comparing the optimized runoff coefficients calculated by this model, the optimization rate of ACO-SCS can be calculated. The SCS-CN model is derived from the water balance equation [76,77]. The model function is as follows:

(1) $$\{\begin{array}{c} Q=\frac{{\left(P-0.2S\right)}^{2}}{P+0.8S},\;P>0.2S\\ Q=0,\;P\leq 0.2 \end{array}$$

where Q is the surface runoff volume (mm), P represents the rainfall volume (mm), and S denotes the potential maximum soil-water capacity, whose function is as follows:

(2) $$S=\frac{25400}{CN}-254$$

where CN is the runoff curve coefficient. The CN is an important parameter for surface runoff simulation of SCS-CN because it is related to many attributes of land, including land use, soil type, and antecedent soil moisture [78]. The CN value is determined by the attached table (Tr-55) in the National Engineering Handbook, Section 4 under certain Antecedent Moisture Conditions (AMC II) [79]. The initial CN can be obtained by combining the land use data, soil data, and hydrological soil grouping data of Guangzhou. Land use data are difficult to characterize the surface pervious differences. Impervious surfaces in urbanization have a great impact on surface runoff [80], which is a method to characterize surface permeability. So the calculation of CN needs to take into account the effects of impervious surfaces [77,81]. The formula of the modified CN is as follows.

(3) $${CN}_{II}=CN+p_{imp}\times \left(98-CN\right)$$

where $CN_{II}$ represents the modified CN under the AMC II conditions and $p_{imp}$ is the density of impervious surfaces. Slope also has a significant effect on surface runoff. If the influence of the slope is ignored, the runoff calculation result will not be accurate for the main urban area of Guangzhou in the hilly area. Therefore, the slope correction method for CN proposed by Williams et al. was used in this study [82]. The modified formula is as follows:

(4) $${CN}_{IIS}=\frac{{CN}_{III}-{CN}_{II}}{3}\times \left[1-2exp\left(-13.86slp\right)\right]+{CN}_{II}$$

(5) $${CN}_{III}={CN}_{II}\times exp[0.00673\times \left(100-{CN}_{II}\right)]$$

where ${CN}_{IIS}$ represents the modified value of ${CN}_{II}$, ${CN}_{II}$, and ${CN}_{III}$ are CN under the AMC II and AMC III, respectively, and slp is considered as the average slope.

#### 2.3.2. Ant Colony Optimization

ACO has the characteristics of a distributed, non-centered control, and distributed individual indirect communication, and is easy to couple with other algorithm models. Due to its excellent ability to solve complex problems in a single individual collaboration, it is widely used to solve optimization problems [83]. Therefore, ACO was used in this study for the optimization of impervious surface space layout with the goal of minimizing the runoff coefficient. The optimization object is the density type of impervious surfaces. To make the optimization results clearer, this study subdivided impervious surfaces into 10 categories with a threshold of 0.1 by referring to Weng et al.’s classification method of impervious surface density: 0–0.2 is classified as extremely low density, 0.2–0.4 is medium-low density, 0.4–0.6 is medium density, 0.6–0.8 is medium-high density, and 0.8–1 is classified as extremely high density [84,85]. The impervious surface density type is encoded with an integer vector of 1–10 (Table 2). An ant represents an optimized layout. The length of each ant is the total number of grid cells. Each position represents a grid cell. A unit value of 1–10 indicates the type of impervious surfaces to be placed.

##### Probability Function

Ants seek the best food path by judging the difference in pheromone concentration at each road node. In ACO, the probability function calculates each node selection probability by combining the pheromone concentration. The probability function is used to measure the ant pheromone of each unit to calculate the probability of each unit selecting an impervious surface type. Since the iterative process requires computing grid cells one by one, the calculation time is relatively long. To simplify the calculation, the product is selected to replace the power exponent by referring to Lu et al.’s practice as follows [86].

(6) $$P_{ij}^{k}=\frac{a\times \tau_{ij,k}+\beta \times \delta_{ij,k}}{\sum_{s={\mathrm{Allowed}}_{s}}\left(a\times \tau_{ij,s}+\beta \times \delta_{ij,s}\right)}\;k=1,2,3,\ldots ,10$$

where ${Allowed}_{s}$ denotes the various impervious surface types that a grid cell can convert, a is a heuristic factor, β is an expecting factor, and $\delta_{ij,k}$ is a heuristic function. The value of β is usually set as the difference of 1 and α. The function $\tau_{ij,k}$ represents the pheromone concentration between the current impervious surface types and the impervious surface types k to be converted to.

##### Heuristic Function

Each ant represents a solution vector for an impervious surface space layout. To avoid falling into the local optimal or global traversal and resulting from excessive dependence on the pheromone concentration, ants must accumulate certain selection experience to improve the accuracy of selection in each component when the impervious surface type is selected. The experience is acquired by setting the heuristic function. In this study, the heuristic function represents the suitability of transforming impervious surface type $k_{1}$ into impervious surface type $k_{2}$. The difference in the runoff coefficient between two impervious surface types was used as the suitability. The formula is as follows:

(7) $$\delta_{ij,k}=\frac{Q_{k_{1}}-Q_{k_{2}}}{P}$$

where $Q_{k_{1}}$ and $Q_{k_{2}}$ stand for the runoff volume of impervious surface type $k_{1}$ and impervious surface type $k_{2}$, respectively, in the grid cell (*i*, *j*), and P is rainfall intensity.

##### The Generation of Ant Colony

**Figure 3:**
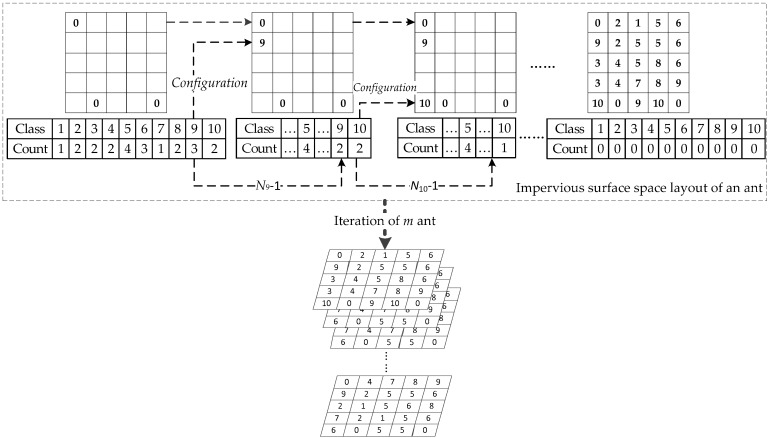
Configuration process of impervious surfaces.

If $P_{ij}^{k}$, the impervious surface type k in grid cell (i,j) with the highest probability, is the same as the current type of impervious surfaces, the type of impervious surfaces in grid cell (i,j) is still k. Otherwise, it is configured according to the principle of a random turnplate. Each impervious surface type *k* is configured to reduce the number of containers corresponding to the impervious surface type *k* by one. ACO-SCS repeats the above process until all grids are configured with impervious surface types. According to such a principle, the impervious surface space layout represented by all ants is configured. The specific process is shown in Figure 3. The number of impervious surface types is *N*.

##### Suitability Evaluation of Ants

To compare the suitability of each ant, it must be measured by a unified evaluation index. In this study, the runoff coefficient of the impervious surface space layout represented by each ant was calculated as the suitability index. The formula is as follows:

(8) $$R_{n}=\frac{\sum_{i}^{r}\sum_{j}^{c}Q_{ij,k}}{num*P}n=1,2,3,\ldots ,ant\text{\_}size$$

where $R_{n}$ indicates the runoff coefficient of the Nth ant, $Q_{ij,k}$ is the runoff volume of impervious surface type k in the grid cell (i,j) by the means of SCS-CN, and num represents the total number of grid cells. If it is in the first iteration, the current optimal solution is the initial spatial layout of impervious surfaces. If not, the minimum runoff coefficient of each generation is compared with the current optimal solution, and impervious surfaces with a smaller runoff coefficient are the optimal solution for this iteration.

##### Update Operation of Pheromone Concentration

Pheromone concentration is an important indicator for ants to determine their pathways. The higher the pheromone concentration, the higher the probability of the ant choosing the path. The ants also leave pheromones after they pass, increasing pheromone concentration. The pheromone concentration of the path chosen by fewer ants will gradually evaporate, and the optimal path will be selected according to this principle. Since the probability of each impervious surface type of each grid cell is required to be recorded, the pheromone concentration matrix is correspondingly set to a 10 *10 *num three-dimensional (3D) matrix. The formula is as follows:

(9) $${Tau}_{t}={Tau}_{t-1}\times \left(1-rho\right)+∆delta$$

where ${Tau}_{t}$ represents the current pheromone concentration, ${Tau}_{t-1}$ denotes the pheromone concentration of the last generation, t is the number of iterations, rho is the volatilization factor of pheromone concentration, and ∆delta indicates the change of the pheromone concentration on each path in this iteration. In this study, the difference in the runoff coefficient between two impervious surface space layouts is used as the increment of each conversion path. Given that the pheromone concentration matrix is set to be a 10 *10 *num 3D matrix, ∆delta is also set to be a 10 *10 *num 3D matrix as follows:

(10) $$∆delta{\left(k_{best},k_{l,t},index\right)}_{l}=\left[∆delta{\left(k_{best},k_{l,t},index\right)}_{l-1}+\left(1-\frac{Q_{l}}{P}\right)\right]\times \sigma \;l=1,2,\ldots ,10;\;index=1,2,\ldots num$$

where index means the location of the grid cell and l indicates the rank of ants after sorting according to the runoff coefficient. Only the top ten ants with the smallest runoff coefficients were selected to update the pheromone concentration. $k_{best}$ represents the impervious surface type configured by the optimal solution at the raster cell index, $k_{l,t}$ indicates the impervious surface type of the Lth ant at the grid cell index, $∆delta{\left(k_{best},k_{l,t},index\right)}_{l}$ is considered as the increment of pheromone concentration at the grid cell index after the update operation of the Lth ant when the impervious surface type $k_{best}$ is converted to $k_{l,t}$, and σ is a constant.

##### Parameter Settings

In the optimization model, some parameters need to be given in advance, including the pheromone volatilization factor, rho, the number of ant colonies, *n*, the heuristic factor, α, the expecting factor, β, and the constant coefficient, σ. By using a test area, the different values of each parameter are set separately. The number of ant colonies is 10–100, the heuristic factor is 0.1–0.95, the expecting factor is the difference between 1 and the heuristic factor, the volatilization factor is 0.1–0.9, and the constant coefficient is 17 values between 0.001–0.8. These data are permutated and combined. To avoid the contingency of the algorithm, ten experiments were carried out for each parameter combination, and the average optimization rate and the average iteration number of each parameter group were recorded when the optimal result was achieved. Finally, 153,010 repeated tests were performed.

**Figure 4:**
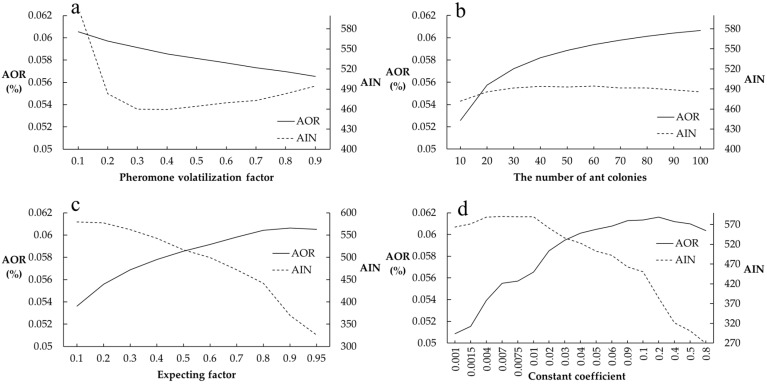
Relationship between parameter setting and average optimization rate (AOR) and average iteration number (AIN): (**a**) pheromone volatilization factor; (**b**) the number of ants; (**c**) expecting factor; (**d**) constant coefficient.

The optimal parameter combination was determined by observing the relationship between each parameter setting and the optimization rate and iteration number. The setting of the volatilization factor is the most important parameter of ACO. When the volatilization factor is too small, the pheromone volatilizes slowly, resulting in a small difference in the pheromone concentration of the different paths, and making it difficult for ants to approach the optimal path. It takes many trials to obtain the best result by traversing. In contrast, if the volatilization factor is too large, the algorithm will easily fall into local optimization. Figure 4a shows the relationship between the volatilization factor, optimization rate, and iteration number. The volatilization factor has a great influence on the optimization rate and iteration number. When the volatilization factor is 0.1, the optimization rate is the highest, but the number of iterations also reaches the largest. When the volatilization factor is 0.9, the optimization rate and iteration number show an opposite trend, and both are small values. Therefore, when setting the volatilization factor, ACO usually tends to select the median value. Considering the optimization rate and iteration number obtained by the experiment, when the volatilization factor is set as 0.4, the optimization rate is close to the optimal value, and the iteration number is the lowest. Therefore, the volatilization factor is set as 0.4 in this study.

Figure 4b shows the influence of the number of ants on the optimization rate and the iteration number. When the number of ants is 10, both the optimization rate and the number of iterations are the minimum values, falling into the local optimum. When the number of ants is more than 70, the optimization rate tends to be stable. The number of ants is positively correlated with the optimization rate. However, the optimization rate tends to be maximized when the number of ants is sufficiently large. In addition, its influence on the iteration number is small. Therefore, the number of ants in this study was set as 70.

Figure 4c shows the expecting factor, and Figure 4d shows the constant coefficient. The effect of the expecting factor and the constant coefficient on the optimization rate and the iteration number shows the same trend. The optimization rate increases with the increasing expecting factor and constant coefficient. When the expecting factor is 0.9, the optimization rate reaches the maximum value and then tends to be stable. Therefore, the expecting factor is selected as 0.9. When the constant coefficient is 0.2, the optimization rate reaches the highest value. The number of iterations decreases as the constant coefficient increases. However, considering that the constant coefficient is the coefficient of the increment of pheromone concentration, if the constant coefficient is too large, it will easily lead to the rapid increase in pheromone concentration in the corresponding path, and the algorithm will fall into local optimization. Therefore, the constant coefficient was set as 0.2 in this study. To sum up, the pheromone volatilization factor, rho, the number of ants, *n*, the expecting factor, β, the heuristic factor, α and the constant coefficient, σ were set as 0.4, 70, 0.9, 0.1, and 0.2, respectively.

###### Integration of Ant Colony Optimization and Soil Conservation Service Curve Number Model

**Figure 5:**
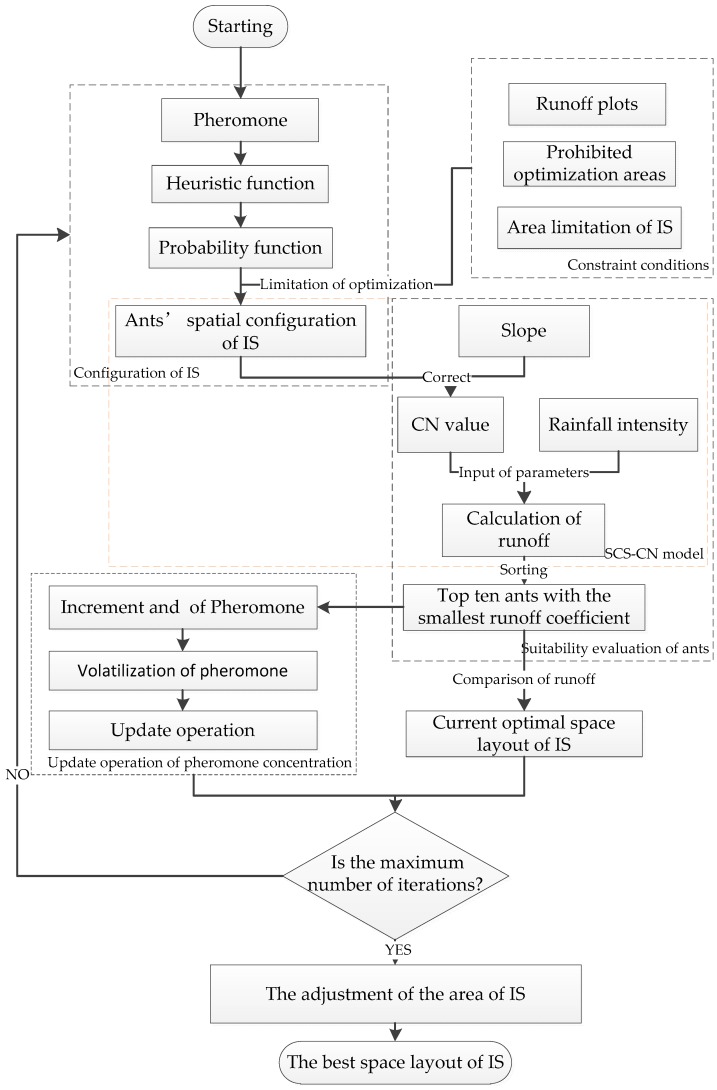
Integration of SCS-CN and ACO. Abbreviation: IS, impervious surfaces.

This study integrates SCS-CN and ACO to optimize the impervious surface space layout. The spatial layout of impervious surfaces is taken as their common parameter, and SCS-CN is set to the objective function of ACO to evaluate the optimization results. The coupling programming of the model and the algorithm is realized by the MATLAB 2014a software (MathWorks, Natick, MA, USA). The main process is as Figure 5:

(1) Set the initial parameters of the algorithm, including the heuristic factor, the expecting factor, the initial pheromone, the volatilization factor, the number of iterations, and the constant coefficient.
(2) Configuration process of impervious surfaces: The heuristic function and the probability function are calculated according to the pheromone. The runoff plot is used as an optimization unit. Each grid cell of each ant was configured with an impervious surface type in turn. Prohibited regions and the number of impervious surface types constrain the optimization regions and areas.
(3) Suitability evaluation of the ants: Impervious surfaces with completed spatial configuration are input as a parameter into SCS-CN to calculate the surface runoff (i.e., the suitability of the ant). The top ten ants with the best suitability were selected. Compared with the optimum impervious surface space layout of the previous iteration, the optimum impervious surface space is selected.
(4) Update operation of pheromone concentration: The increment and volatility of pheromones were calculated according to the top ten ants with the current optimum suitability, and then the pheromone concentration was updated.
(5) Condition for stopping the algorithm: If the current iteration number reaches the maximum iteration number, the algorithm ends. The current optimal space layout of impervious surfaces is the final result. Instead, repeat step 2 until the maximum number of iterations is reached. When iteration stops, runoff coefficients before and after optimization are calculated by the SCS-CN model, and the optimization rate is obtained by comparison.
(6) Adjustment of the area of impervious surface: First, the increment of the runoff coefficient of each grid element after iteration is calculated and sorted. Second, the impervious surface type corresponding to the grid cell with the largest increment of runoff coefficient remains unchanged. The adjusted impervious surface area is calculated and compared to the initial impervious area. If the result is not lower than the limited area, the adjusted impervious surfaces repeat the above operation until the area is closest to the standard.

**Figure 6:**
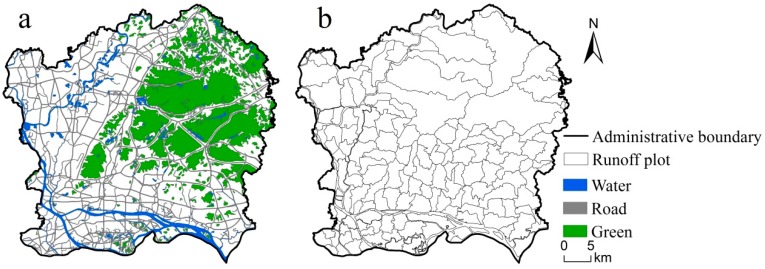
The prohibited optimization areas: (**a**) prohibited optimization areas; (**b**) urban runoff plots.

In the process of optimization, some specific areas are limited, taking into account factors such as ecological environmental protection and urban planning and development. These areas mainly include large water bodies (i.e., lakes and rivers), roads (urban arterial roads, highways, and expressways), and large green spaces (parks and woodlands). As shown in Figure 6a, these areas are used as masks to erase the study area.

The urban runoff plot was divided by combing the urban watershed division technique with the detail control planning area index, which realized the combination of hydrological and ecological units with traditional planning units. Such a combination makes the urban runoff plot more natural and ecologically significant and simultaneously considers the urban-rural planning significance [29]. Therefore, the runoff plot is taken as the optimization unit in this study (Figure 6b), which meets the requirements of the urban planning unit. Optimization was only carried out in the runoff plot to reduce the update difference before and after optimization. The city is an important area of life and production. Considering that urban renewal cannot lower the quality of living and production, the area of construction land cannot be significantly reduced. Therefore, before spatial optimization, the number of impervious surface types in the runoff plot was first counted and then extracted one by one in the process of space allocation to ensure that the impervious surface area meets the constraint conditions after optimization.

### 2.4. Landscape Pattern Index

The landscape pattern index is a simple quantitative index that can highly concentrate landscape pattern information and reflect its structural composition and spatial configuration. This index can be used to conduct quantitative research on the composition, spatial configuration, and dynamic change of landscapes [87,88]. Since the landscape pattern index is an effective tool for quantifying the structure and pattern of thematic maps and helps explain the urban spatial structure, it has gradually been applied to urban environmental research. Therefore, to compare the spatial layout differences of impervious surfaces before and after optimization, the landscape pattern index under the scale of patch class was used to study its changes. The specific landscape pattern index is shown in Table 3.

## 3. Results

### 3.1. Comparison of Impervious Surface Changes after Optimization

**Figure 7:**
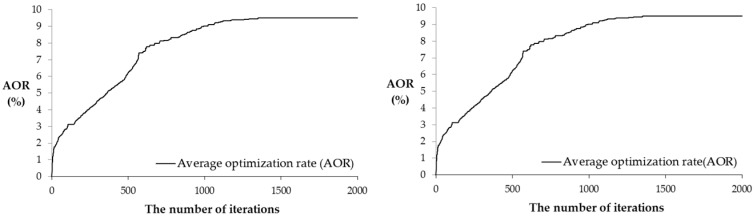
Model optimal curve.

ACO-SCS aims to minimize the runoff coefficient and is used to optimize the impervious surface space layout of the research area. As shown in Figure 7, the number of iterations is set to 2000 times. In the initial stage, the average optimization rate increased rapidly but gradually slowed as the number of iterations increased and finally reached the highest value at approximately 1400 times. This indicates that the surface runoff coefficient is the minimum under the condition of the current impervious surface space layout.

**Figure 8:**
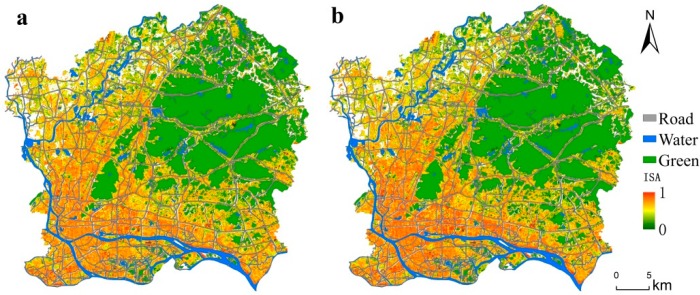
Impervious surface spatial distribution before and after optimization: (**a**) impervious surface spatial distribution before optimization and (**b**) impervious surface spatial distribution after optimization.

**Figure 9:**
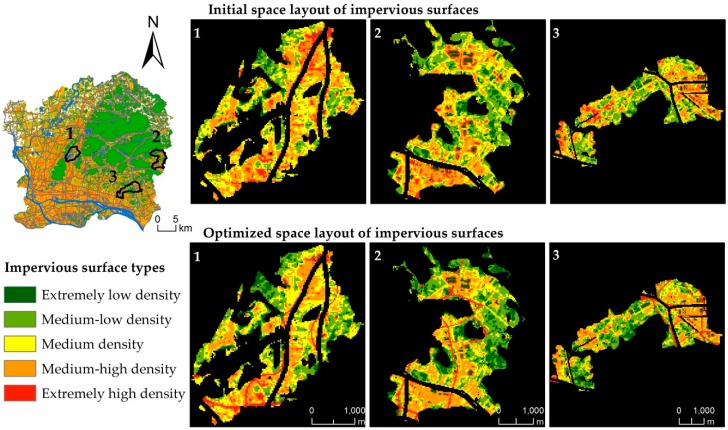
The demonstration of optimal results in runoff plot scale.

The optimized impervious surface space layout is shown in Figure 8. Water bodies, green spaces, and urban arterial roads are prohibited to be optimized. The water bodies are mainly the Pearl River, reservoirs, and large ponds in the city, while the green spaces are mainly the northeast region, such as Baiyun Mountain and Maofeng Mountain. Macroscopically, the impervious surface space layout has not changed significantly. The high-density impervious surfaces are still preserved in the central urban areas, such as Tianhe, Yuexiu, Liwan, and Haizhu, mainly because this study uses a runoff plot as the optimization unit and the impervious surfaces in each runoff plot are optimized independently. Additionally, the spatial distribution of impervious surfaces within the runoff plot varies greatly before and after optimization. To observe the optimal results of the impervious surface space layout from the scale of the runoff plot, three runoff plots with high optimization rates and global distributions were selected for display from the perspective of the optimization efficiency and the spatial balance of runoff plots. The effect is shown in Figure 9. In terms of spatial layout, extremely low-density, medium-low density, and medium-high density impervious surfaces are more clustered, while extremely high-density impervious surfaces are shown as long strips of agglomeration. Medium density impervious surfaces become more fragmented.

**Figure 10:**
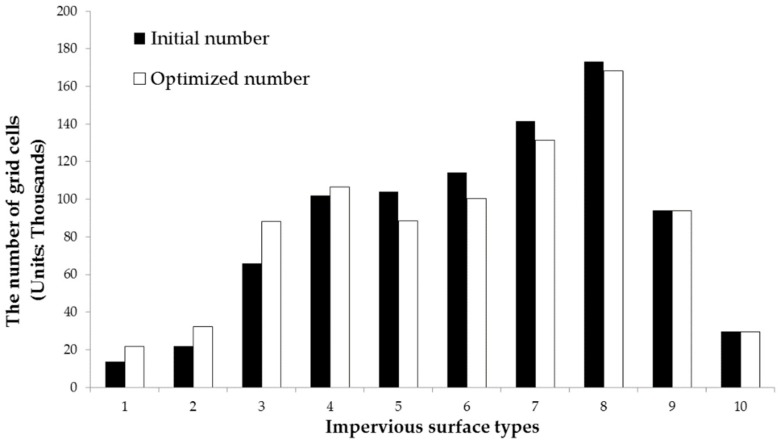
Statistics of impervious surface type before and after optimization.

**Figure 11:**
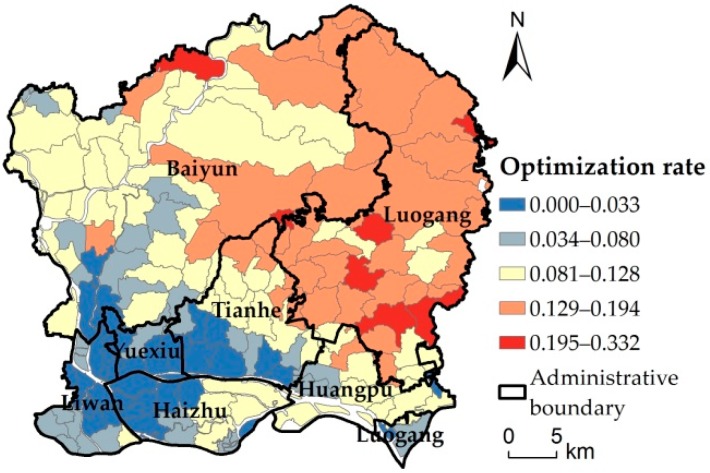
Statistics of impervious surface type before and after optimization.

By counting the changes in the number of impervious surface types (Figure 10), the number of impervious surface types of medium-low density and extremely low density was on the rise, while the number of impervious surface types of medium and medium-high density was on the decline. Specifically, impervious surface types 5, 6, and 7 decreased, while the extremely high density impervious surfaces did not change significantly. In general, the average impervious surface density decreased from 0.5765 to 0.5572, consistent with the 5% variation range of impervious surfaces. Under the annual rainfall intensity, the average optimization efficiency can reach 9.52%. The highest optimization rate was 33.16%, and the number of runoff plots with an optimization rate over 10% was 67, with a good optimization effect. In terms of spatial distribution of optimization rate, the spatial heterogeneity of the optimized results was found from the demonstration of the optimized results in each runoff plot (Figure 11). The model optimization effect achieves the best effect in the urban fringe with low urban construction land density, while the optimization effect is not obvious in the urban core areas with high construction land.

### 3.2. Landscape Pattern Change

**Figure 12:**
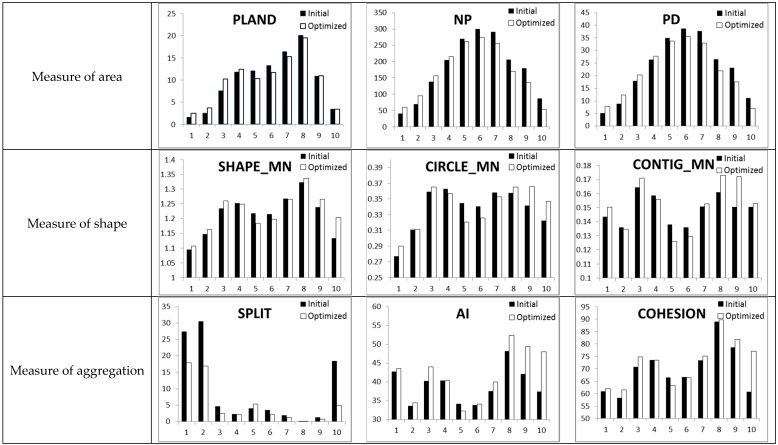
Landscape pattern index statistics under patch class scale: the percent of landscape (PLAND), number of patches (NP), patch density (PD), mean shape index (SHAPE_MN), mean related circumscribing circle (CIRCLE_MN), mean contiguity index (CONTIG_MN), aggregation index (AI), splitting index (SPLIT), and patch cohesion index (COHESION).

Under the scale of patch class, the landscape pattern index of impervious surfaces before and after optimization was calculated from the three measures of area, shape, and aggregation (Figure 12). On the measure of area, the percent of landscape (PLAND) was consistent with the change in impervious surface types. The number of impervious surface types of medium-low density and extremely low density was on the rise, while the number of impervious surface types of medium and medium-high density was on the decline. Specifically, impervious surface types 5, 6, and 7 decreased, while the extremely high density impervious surfaces did not change significantly. For the number of patches (NP), extremely low and medium-low density impervious surface patch classes increased. Conversely, medium, medium-high, and extremely highdensity impervious surface patch classes declined. As a result, the change trend of patch density (PD) was consistent with that of the number of patches (NP). However, the impervious surface type of the middle density after optimization is still the main proportion, and the extremely low-density and extremely high density impervious surface proportion are still the lowest.

In terms of the shape measure, impervious surface types of extremely low, medium-low, medium-high, and extremely high density show an increasing trend for the mean shape index (SHAPE_MN). This indicates that the shapes of these impervious surface types tend to be complicated. The medium-density impervious surface type shows a downward trend. This indicates that the shape of the impervious surface patch is becoming simpler. However, the types of impervious surface patches 4 and 7 did not change significantly. For the mean related circumscribing circle (CIRCLE_MN), the change trend is consistent with that of the mean shape index (SHAPE_MN). The impervious surface patches with extremely low, medium-low, medium-high, and extremely high densities increased. The mean contiguity index (CONTIG_MN) of these impervious surface types also generally increased. This indicates that their inner adjacency degree and connectivity increased, but their shape became flatter and longer. In contrast, the medium-density impervious surface type shows a decrease in both indexes. This indicates that the connectedness of the impervious surface type decreases in a curved, coiled but narrow-hollow form.

In terms of the aggregation measure, impervious surface patch classes before and after optimization are consistent in the splitting index (SPLIT), the aggregation index (AI), and the patch cohesion index (COHESION). The impervious surface patches with extremely low, medium-low, medium-high, and extremely high densities have reduced fragmentation and increased spatial connectivity and aggregation. In addition, medium-density impervious surfaces show an opposite trend.

In general, the impervious surfaces of medium and medium-high density decreased in quantity after the optimization. Extremely low and medium-low density impervious surfaces increased, and the number of extremely high densities remained the same. The shape of the impervious surface types of extremely low, medium-low, medium-high, and extremely high densities are more complex, but the degree of fragmentation is reduced, and the space gathering is stronger. However, impervious surfaces of medium density have an opposite trend. The shape was more curved and coiled, the phenomenon of hollowing increased, the degree of fragmentation increased, and the spatial connectivity decreased. Combining with the optimization results of runoff plot scale, it is found that the construction of a high–low–high density discontinuous connection of impervious surfaces is helpful to reduce runoff. The connection mode can be achieved by transforming the medium-density impervious surfaces into low-density impervious surfaces in quantitative terms and constructing the agglomeration of low-density impervious surfaces and the oblong agglomeration of high-density impervious surfaces in the space layout.

## 4. Discussion

### 4.1. Evaluation of Optimization Model

At present, many scholars have proven that the practice of low-impact development technology can effectively reduce surface runoff. Zhang et al. simulated surface runoff using three methods (green roof, permeable surface, and rainwater bucket device) for low-impact development. The results showed that the green roof reduced runoff by 1.7%–2.0%, the permeable surface by 0.6%–8.4%, and the rainwater bucket device by 7.1%–36.8% [89]. Ahiablame et al. demonstrated that the optimization effects of the three methods of green roof, permeable surface and the rainwater bucket device were 11%–39%, 5%–19%, and 3%–13%, respectively [90]. Mentens et al. reduced runoff by 2.7% by converting 10% of impervious roofs to green roofs [91]. From the perspective of spatial layout optimization of impervious surfaces, this research model reduces the impact of urban development on surface runoff. The results show that the average optimization rate is 9.52% and the maximum optimization rate reaches 33.16%. Compared with the existing research results, the optimal results of this study are generally close to the simulation effect of low-impact development technology. The average optimization rate is better than that of the green roof. Therefore, it can be considered that the integration of SCS-CN and ACO supported by geographic simulation technology has a good optimization effect.

It is not advisable for some cities with stable development to reduce the impact of rapid urbanization on urban waterlogging through large-scale urban reconstruction activities. This practice not only changes the original urban landscape pattern, but also costs too much to bear [34,35,36,37]. Therefore, this study aims to optimize the space layout of impervious surfaces and reduce the optimization cost under the premise of protecting the existing impervious surface density as much as possible. In addition, urban green space, water bodies, and urban arterial roads are important components of urban landscape ecological patterns. By setting the above areas as forbidden patches, the damage to urban ecological landscapes can be effectively reduced, and the operability of the model optimal results can be increased. In urban development, urban planning activities are carried out in planning units. To make the spatial optimization of impervious surfaces meet the requirements of urban planning as much as possible, the optimization unit is divided strictly according to the standard area of the urban planning unit. In summary, the optimal results of the model are good, and the results are operable and pertinent, which can better provide a policy-making reference for urban renewal.

### 4.2. The Influence of Various Factors on the Optimization of Impervious Surface Space Layout

#### 4.2.1. The Influence of Input Factors on the Optimization of Impervious Surface Space Layout

**Figure 13:**
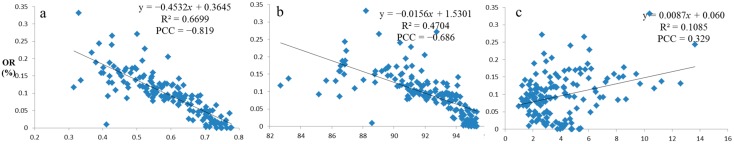
Regression modeling of optimization rate and input factors: (**a**) initial impervious surfaces; (**b**) CN value; and (**c**) slope.

In the running of the model, each input element will affect the final optimization result. Therefore, to understand the key factors affecting the model, the influences of various input factors on the model results are first studied. The input factors of the optimization model mainly include the mean of the initial impervious surfaces, the mean of the CN value, and the mean of the slope in the study area. To understand its influence on optimal results, a regression model was established for the input factors and the optimization rate of 154 runoff plots, and the Pearson correlation coefficient (PCC) was calculated. As shown in Figure 13, the optimization rate builds a linear regression model with the initial impervious surface density, CN value, and slope. The slope of the regression model was −0.4532, −0.0.0156, and −0.0087, and the variance was 0.67, 0.47, and 0.11, respectively. At the significance level of 0.01, the PCC between the optimization rate and the initial impervious surface density is the strongest at −0.819, followed by that of the CN value, and that of the slope is the weakest at 0.329. The initial impervious surface density and CN value are negatively correlated with the optimization rate, while the slope is positively correlated. The slope has the weakest influence on the optimization rate. The reason is that except for the main mountains of the Baiyun Mountain and the Maofeng Mountain in the northeast, which are the prohibited optimization areas, the rest of the urban areas is hilly basins. Although there are differences in the slopes of the various runoff communities in these areas, the fluctuations are not large. Only five runoff plots had mean slopes of more than 10. Therefore, the correlation between the optimization rate and the slope is weak. The optimization rate is negatively correlated with the CN value. In SCS-CN, the CN value is the only input parameter. The CN value in the model is the CN value corrected by the impervious surfaces and slope, which is closer to the actual value. Therefore, the higher the initial CN value is, the smaller the correction effect of the impervious surfaces and slope will be. Therefore, no matter how the impervious surface space layout changes, the correction effect on the CN value will be small, resulting in a low optimization rate. In contrast, in the runoff plot with a low initial CN value, where the correction effect of the impervious surfaces and slope is large, the change in the impervious surface space layout also has a great influence on the corrected CN value. Thus, it improved the possibility of obtaining a better optimization effect. For impervious surface density, when the average density of the impervious surfaces is large, the impervious surface density of each unit is high. As a result, under the limitation of impervious surface density change, the impervious surface density of each unit still maintains a high value after the spatial layout optimization of the impervious surfaces. Thus, the spatial layout with large differences cannot be generated, resulting in small differences in runoff coefficients before and after optimization. In contrast, when the mean density of the impervious surfaces is low or medium, there are more kinds of impervious surfaces to be selected, resulting in a spatial layout with great difference. Therefore, this kind of impervious surface provides the possibility of reducing the runoff coefficient and obtaining a better optimization effect. As shown in Figure 13a, although the average density of the impervious surfaces is the same, it still produces a great difference in the optimization effect. This may have something to do with the spatial layout of the initial which are the impervious surfaces. Therefore, it is necessary to explore the relationship between the optimization rate and the spatial layout of the initial impervious surfaces.

#### 4.2.2. The Influence of Initial Impervious Surface Space Layout on the Optimization Model

##### Relationship between Optimization Rate and Proportion of Impervious Surface Type

**Figure 14:**
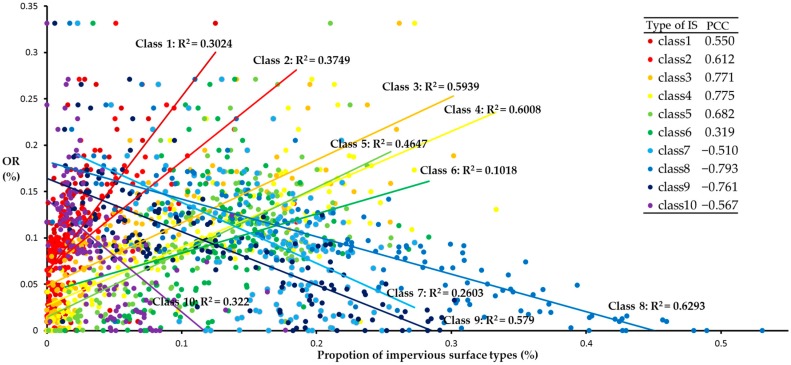
Regression modeling between optimization rate and proportion of impervious surface type. Abbreviations: IS, impervious surfaces; OR, odds ratio.

The above discussion has shown that there is a strong negative correlation between the model optimization rate and the initial impervious surface density. To further understand the specific relationship between the optimization rate and the impervious surface types, this study obtained the relationship between the optimization rate and various types of impervious surfaces and conducted linear regression modeling. The results are shown in Figure 14. From the perspective of slope, the optimization rate is positively correlated with the proportion of impervious surface types 1–6 and negatively correlated with types 7–10. The slope cannot be directly compared for the significant difference in regression variances being affected by the proportion of each type of initial impervious surfaces, so the PCC is calculated. According to the results of PCC at the significance level of 0.01, it can be divided into three levels. The first level is impervious surface type 6. PCC and regression modeling have the lowest effect. This indicates that the relationship between the optimization rate and impervious surface type 6 is the weakest. The second level includes impervious surface types 1, 2, 5, 7, and 10. The PCC and regression modeling effect are at a medium level. The relationship between the optimization rate and these impervious surface types is enhanced. The impervious surface types at the third level are 3, 4, 8, and 9, which have the strongest correlation with the optimization rate and the best regression modeling effect. This indicates that the impervious surface types 3, 4, 8, and 9 have the greatest influence on the optimization effect in terms of the proportion of impervious surface types. Moreover, the optimization rate is positively correlated with impervious surface types 3 and 4, and negatively correlated with impervious surface types 8 and 9. It also exactly confirms that the lower the average density of the impervious surfaces, the higher the optimization rate. Only when impervious surface types 3 and 4 account for a relatively high level, is there a possibility that a more widely different impervious surface space layout is produced. Therefore, the proportion of impervious surface types 3, 4, 8, and 9 is a key factor in the optimization model.

##### Relationship Between Optimization Rate and Space Layout of the Initial Impervious Surfaces

**Figure 15:**
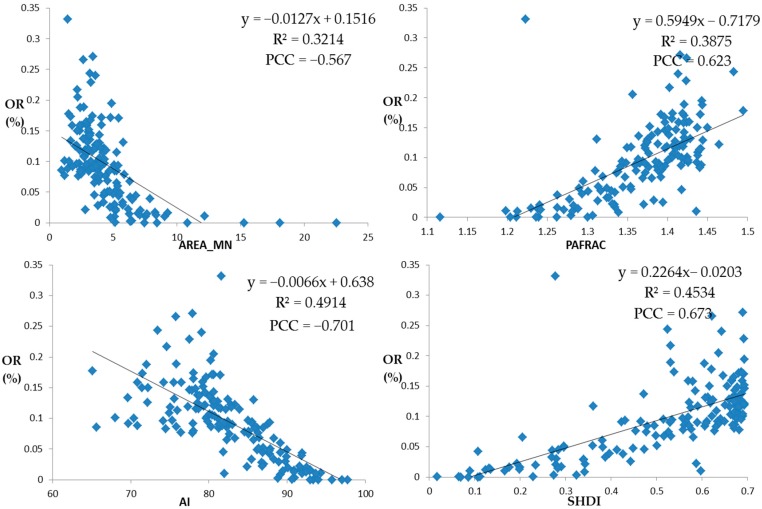
Regression modeling of optimization rate and landscape pattern index: (**a**) area-weighted mean of patch area (AREA_MN); (**b**) perimeter-area fractal dimension (PAFRAC); (**c**) aggregation index (AI); (**d**) Shannon’s diversity index (SHDI). Abbreviation: PCC, Pearson correlation coefficient.

In addition to the influence of the proportion of impervious surface types on the optimization rate, the initial spatial layout of the impervious surfaces also has a great influence on the optimization rate. From the scale of landscape, the most correlated and representative landscape pattern index among the four measures of area, shape, aggregation, and diversity was selected for regression modeling, and the results are shown in Figure 15.

On the area measure, the area-weighted mean of the patch area was selected for regression modeling with the optimization rate. The optimization rate was negatively correlated with the area-weighted mean of the patch area. This indicates that the more patches there are on the initial impervious surfaces, the greater the patch density will be, and the better the model optimization effect will be. On the shape measure, the perimeter-area fractal dimension was selected for regression modeling with the optimization rate. The optimization rate was positively correlated with the perimeter-area fractal dimension. The results show that the more complex the initial patch shape is, the better the model optimization effect will be. On the aggregation measure, the aggregation index was selected for regression modeling with the optimization rate. The optimization rate was negatively correlated with the aggregation index. This indicates that the more fragmented the initial impervious surfaces are, the higher the degree of fragmentation is, and the better the model optimization effect will be. On the diversity measure, Shannon’s diversity index was selected for regression modeling with the optimization rate. It was found that the optimization rate was positively correlated with Shannon’s diversity index. This indicates that the richer the initial impervious surface types are, the greater the difference will be, and the better the model optimization effect will be.

In other words, the initial impervious surface space layout has an important impact on the optimal results of the model. The more diverse the type of the impervious surface space layout is, the more complex the shape, the more discrete the patch, and the better the model application effect will be. Therefore, for urban fringes with diverse types of impervious surfaces, complex shapes of impervious surfaces and high dispersion, the effect of reducing surface runoff by optimizing the spatial layout of the impervious surfaces is more significant.

### 4.3. Practical Significance of Optimal results

Presently, the development of Guangzhou has shifted from an increase in urban scale to the improvement of urban quality. How to realize the efficient promotion of urban land inventory is a major problem facing urban renewal. The improvement of land quality will be the key to urban renewal for urban rainstorm waterlogging prevention and control. The optimal results provide an important reference for urban renewal planning.

**Figure 16:**
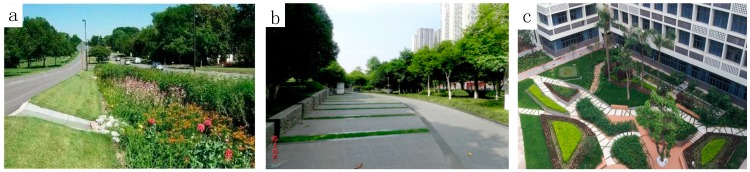
The instance of optimization results in low-impact development (LID): (**a**) concave herbaceous field; (**b**) sunken green space square; and (**c**) grid construction inside the campus.

Combining the above analysis, to reduce the reconstruction cost of impervious surfaces on the premise of protecting the existing impervious surfaces to the maximum extent in the actual situation, the connection difference of the impervious surfaces should be increased, and the gradual-change connection mode of the impervious surface density should be reduced. By means of high–low–high density connection of impervious surfaces, surface runoff can be reduced. This optimization method of impervious space layout is similar to the concept of LID landscape construction (Figure 16). This practice of high–low impervious surfaces connection includes concave herbaceous fields beside the road, sunken green space squares, and grid construction inside the campus [92,93,94]. The impervious surface layout of high–low–high density discontinuous connection is constructed through the flat and long aggregation of low-density and high-density impervious surfaces. This method helps to break the connectivity of high-density impervious surfaces and to prevent the confluence of surface runoff on the high-density impervious surfaces, thus reducing the impact of impervious surfaces on surface runoff.

Due to the spatial heterogeneity of optimization efficiency in urban suburbs and urban core areas, when making the urban renewal planning decision for the prevention and control of urban rainstorms and waterlogging, more pertinence should be considered. For areas such as Baiyun, Luogang, and Tianhe North, LID in common urban planning, such as concave herbaceous fields, sunken green space squares, and grid construction can be adopted. However, for areas such as the south of Tianhe, Liwan, and the west of Haizhu, the underground pipe network, slope, river channel, urban infiltration surface, and other factors should be considered in the policy-making process.

## 5. Conclusions

Currently, urban rainstorm waterlogging has seriously affected urban sustainable development. Optimizing the design of impervious surfaces in urban renewal planning to reduce its impact on surface runoff is an important way to prevent and control urban waterlogging. Based on the geographical simulation framework, this study integrated SCS-CN and ACO to optimize impervious surface space layout to minimize surface runoff. It provides a decision-making basis for urban renewal planning for urban rainstorm waterlogging prevention and control and follows the trend of urban development mode from the scale expansion of incremental land to the quality improvement of inventory land.

The ACO-SCS model based on geographic simulation technology to optimize the spatial layout of impervious surfaces can effectively reduce the aggravation of urban rainstorm waterlogging caused by urbanization. The optimization effect of the model is good and stable, with an average optimization rate of 9.52% and a maximum optimization rate of 33.16%. The model is affected by many factors. In addition to the influence of the input parameters on the model, the initial spatial layout of the impervious surfaces is the key influencing factor. The optimization model with impervious surfaces of high proportion of medium-low density type, complicated shape, and high dispersion is more likely to obtain better results.

The optimal results can provide a reference for urban renewal planning. The results show that the spatial layout optimization method of “high–low–high” impervious surface connection can effectively reduce the impact of urbanization on surface runoff by transforming the impervious surface type of the medium density into that of the low density on the scale of urban runoff plots. However, there is spatial heterogeneity in the optimal results, and the optimization effect of the urban fringe is better. Therefore, urban renewal planning for urban rainstorm waterlogging prevention and control should be different. For urban renewal planning in urban fringes, the optimal results can be referenced to a large extent. The spatial layout of the impervious surfaces can be optimized to alleviate urban rainstorm waterlogging. For urban renewal planning in urban core areas, more factors affecting waterlogging should be considered comprehensively for decision-making and exploration.

This coupling parameter, depending on the input and output of data, can ensure the independence between the model and algorithm and improve the stability of the program. However, due to the characteristics of SCS-CN based on grid element calculation, the model needs to conduct an impervious surface type configuration for each grid cell in the study area. This causes the model to run for a long time. Therefore, the comparison and optimization of the coupling model will be the key research direction in the future. Although impervious surfaces are the key factor in urban rainstorm waterlogging, many research results show that the underground pipe network, river channels, slope, and rainwater management methods all have important influences. Therefore, the comprehensive consideration of these influencing factors in the optimization model will be an important breakthrough in follow-up research to better serve the decision-making in urban renewal planning.

## Figures and Tables

**Table 1:** Metadata information.

Data	Format	Time	Source
1:4 Million administrative divisions	Esri shapefile	2005	National Geomatics Center of China
Landsat remote-sensing image	Img	28-10-2010	United States Geological Survey
High-resolution remote sensing image	Jpeg	2010	Google Earth satellite imagery
ASTER GDEM	Img	2009	Land Processes Distributed Active Archive Center
Global soil classification standard data	Grid	2009	WestDC China
Land use data	Esri shapefile	2010	The second national land survey in Guangzhou
Urban runoff plots	Esri shapefile	2009	Study results of Li et al. [74]

**Table 2:** Classification of the density type of impervious surfaces.

**Impervious Surface Density**	0–0.1	0.1–0.2	0.2–0.3	0.3–0.4	0.4–0.5	0.5–0.6	0.6–0.7	0.7–0.8	0.8–0.9	0.9–1
**Type Encoding**	1	2	3	4	5	6	7	8	9	10
**The Type of Impervious Surfaces**	extremely low density		medium-low density		medium density		medium-high density		extremely high density	

**Table 3:** Landscape pattern index.

Landscape Pattern Index		Unit	Value Range
Measure of area	Percent of landscape (PLAND)	%	0–100
	Number of patches (NP)	pcs	>0
	Patch density (PD)	pcs/100 hm^2^	>0
Measure of shape	Mean shape index (SHAPE_MN)	no	≥1
	Mean related circumscribing circle (CIRCLE_MN)	no	0–1
	Mean contiguity index (CONTIG_MN)	no	0–1
Measure of aggregation	Aggregation index (AI)	%	0–100
	Splitting index (SPLIT)	no	≥1
	Patch cohesion index (COHESION)	no	≥0
